# Supplementary material for: MetaRibo-Seq measures translation in microbiomes
Source: Nat Commun. 2020 Jun 29;11:3268. doi: 10.1038/s41467-020-17081-z (PMC7324362; doi:10.1038/s41467-020-17081-z)
Supplement: Supplementary file 10 — Supplementary Data 7 [file 41467_2020_17081_MOESM10_ESM.zip › File2/Confidence_High_Taxonomy/357223_out.krona.html]

Javascript must be enabled to view this page.

members
magnitude
magnitudeUnassigned
count
unassigned
taxon
rank

357223\_out

200

superkingdom
200
2

200
976
phylum

200643
200
class

order
200
171549

family
14
815

2
816

SRS013098\_contig\_number\_40871SRS050925\_contig\_number\_14330
14
genus

1
2292944
species

SRS077849\_contig\_number\_19416

46506
1

SRS105153\_contig\_number\_15472
species

1
820
species

SRS1055022\_contig\_number\_18726

821
6
species

SRS011405\_contig\_number\_15119SRS017521\_contig\_number\_38853SRS019381\_contig\_number\_8228SRS019968\_contig\_number\_10111SRS098571\_contig\_number\_64812SRS103987\_contig\_number\_28908

species

SRS050026\_contig\_number\_6832SRS098061\_contig\_number\_4615
2
28116

246787
1

SRS1041118\_contig\_number\_11504
species

family
1
171552

577309
1
genus


SRS048164\_contig\_number\_31300
species
1
454154

family
171550
1

genus
239759
1

1946009
1

SRS140492\_contig\_number\_11457
species

180
2005525
family

genus
180

SRS019178\_contig\_number\_1129SRS063928\_contig\_number\_657SRS1055076\_contig\_number\_4851SRS142890\_contig\_number\_40782
4
375288

152
46503
species

SRS011302\_contig\_number\_26659SRS012969\_contig\_number\_34486SRS013476\_contig\_number\_20746SRS013521\_contig\_number\_9477SRS013687\_contig\_number\_34236SRS013800\_contig\_number\_1852SRS013940\_contig\_number\_17271SRS013951\_contig\_number\_34108SRS014287\_contig\_number\_10487SRS014412\_contig\_number\_2971SRS014459\_contig\_number\_29059SRS014613\_contig\_number\_13195SRS014683\_contig\_number\_14562SRS015217\_contig\_number\_29336SRS015264\_contig\_number\_10681SRS015264\_contig\_number\_19921SRS015694\_contig\_number\_14852SRS015782\_contig\_number\_contig-100\_490.195236SRS016056\_contig\_number\_1601SRS016132\_contig\_number\_1423SRS016381\_contig\_number\_159SRS016753\_contig\_number\_824SRS016840\_contig\_number\_1686SRS017247\_contig\_number\_2936SRS017433\_contig\_number\_2788SRS018049\_contig\_number\_1730SRS018888\_contig\_number\_6341SRS019030\_contig\_number\_10831SRS019068\_contig\_number\_100597SRS019161\_contig\_number\_1609SRS019286\_contig\_number\_contig-100\_386.35422SRS019397\_contig\_number\_1923SRS019445\_contig\_number\_17767SRS019685\_contig\_number\_19035SRS019808\_contig\_number\_19577SRS019910\_contig\_number\_1691SRS020271\_contig\_number\_1356SRS022137\_contig\_number\_11332SRS022524\_contig\_number\_15273SRS023526\_contig\_number\_20470SRS023583\_contig\_number\_16846SRS023914\_contig\_number\_22839SRS024009\_contig\_number\_3036SRS024075\_contig\_number\_3788SRS024132\_contig\_number\_12496SRS024435\_contig\_number\_22934SRS043841\_contig\_number\_8559SRS044535\_contig\_number\_13869SRS045244\_contig\_number\_8428SRS045713\_contig\_number\_12102SRS045739\_contig\_number\_8971SRS046068\_contig\_number\_1058SRS046717\_contig\_number\_2253SRS047044\_contig\_number\_17828SRS047433\_contig\_number\_11143SRS048981\_contig\_number\_3319SRS049402\_contig\_number\_10804SRS049712\_contig\_number\_18317SRS049959\_contig\_number\_42821SRS049995\_contig\_number\_17451SRS050299\_contig\_number\_3501SRS050520\_contig\_number\_13SRS050941\_contig\_number\_20248SRS051031\_contig\_number\_28544SRS051610\_contig\_number\_3481SRS051882\_contig\_number\_contig-100\_447.107648SRS052078\_contig\_number\_2957SRS052697\_contig\_number\_34265SRS053356\_contig\_number\_23917SRS055966\_contig\_number\_805SRS055982\_contig\_number\_4028SRS058770\_contig\_number\_1563SRS062427\_contig\_number\_4676SRS062654\_contig\_number\_21258SRS063040\_contig\_number\_22651SRS063127\_contig\_number\_797SRS063489\_contig\_number\_2423SRS063489\_contig\_number\_2423SRS063985\_contig\_number\_20325SRS064232\_contig\_number\_262SRS064276\_contig\_number\_4169SRS064757\_contig\_number\_16745SRS065504\_contig\_number\_13227SRS074670\_contig\_number\_4671SRS075021\_contig\_number\_2805SRS075821\_contig\_number\_21387SRS075984\_contig\_number\_26119SRS076804\_contig\_number\_18411SRS077086\_contig\_number\_6634SRS077552\_contig\_number\_6733SRS077589\_contig\_number\_845SRS078176\_contig\_number\_9870SRS078242\_contig\_number\_27420SRS078419\_contig\_number\_35317SRS078665\_contig\_number\_2130SRS097889\_contig\_number\_38767SRS097920\_contig\_number\_27285SRS097958\_contig\_number\_21818SRS098644\_contig\_number\_33854SRS098655\_contig\_number\_12297SRS101433\_contig\_number\_1779SRS104036\_contig\_number\_8254SRS1041031\_contig\_number\_20755SRS1041039\_contig\_number\_13345SRS1041091\_contig\_number\_20293SRS1041092\_contig\_number\_contig-100\_1524.76683SRS1041112\_contig\_number\_1247SRS1041130\_contig\_number\_1097SRS1041134\_contig\_number\_13773SRS1041137\_contig\_number\_23684SRS1041139\_contig\_number\_518SRS1041140\_contig\_number\_11212SRS1041141\_contig\_number\_6155SRS104327\_contig\_number\_2366SRS104636\_contig\_number\_26074SRS104912\_contig\_number\_15940SRS105082\_contig\_number\_10483SRS1054716\_contig\_number\_12200SRS1055069\_contig\_number\_8082SRS140513\_contig\_number\_10825SRS142503\_contig\_number\_37719SRS142923\_contig\_number\_contig-100\_2094.114466SRS142980\_contig\_number\_950SRS143148\_contig\_number\_21987SRS143181\_contig\_number\_11741SRS143181\_contig\_number\_contig-100\_158.83718SRS143372\_contig\_number\_9561SRS143780\_contig\_number\_5734SRS143876\_contig\_number\_40918SRS143991\_contig\_number\_18048SRS144297\_contig\_number\_14502SRS144506\_contig\_number\_28514SRS144714\_contig\_number\_28561SRS145308\_contig\_number\_2113SRS146764\_contig\_number\_1415SRS146813\_contig\_number\_29769SRS147346\_contig\_number\_42581SRS147425\_contig\_number\_1478SRS147557\_contig\_number\_25526SRS147653\_contig\_number\_1396SRS148091\_contig\_number\_9419SRS148159\_contig\_number\_26130SRS148253\_contig\_number\_8645SRS148784\_contig\_number\_9908SRS148817\_contig\_number\_contig-100\_459.79926SRS149075\_contig\_number\_13076SRS149181\_contig\_number\_11835SRS893230\_contig\_number\_6806SRS893292\_contig\_number\_764SRS893295\_contig\_number\_9030SRS893300\_contig\_number\_1074SRS893327\_contig\_number\_3961

823
1

SRS015065\_contig\_number\_20262
species

species

SRS143070\_contig\_number\_12775
1
1750560


SRS014948\_contig\_number\_23700SRS017821\_contig\_number\_30876SRS017859\_contig\_number\_4905SRS018133\_contig\_number\_8276SRS046369\_contig\_number\_9728SRS048870\_contig\_number\_12382SRS049896\_contig\_number\_30507SRS050422\_contig\_number\_36186SRS058070\_contig\_number\_19505SRS065176\_contig\_number\_14390SRS075341\_contig\_number\_11378SRS075773\_contig\_number\_36034SRS076876\_contig\_number\_463SRS1041136\_contig\_number\_11154SRS143722\_contig\_number\_14920SRS147271\_contig\_number\_19975SRS148196\_contig\_number\_24989SRS148319\_contig\_number\_3608SRS893170\_contig\_number\_8547SRS893252\_contig\_number\_10208SRS893382\_contig\_number\_2321
species
21
1263094


SRS144135\_contig\_number\_32922
species
1
1263091

4
1853231
family

genus
2
574697

species

SRS015663\_contig\_number\_13557SRS020233\_contig\_number\_40643
2
544645

genus
2
283168

28118
2
species

SRS020328\_contig\_number\_27544SRS053398\_contig\_number\_11357
